# Research Review on Inhibitors of Lumpy Skin Disease Virus: From Biological Characteristics, Drug Repurposing, to Computer‐Aided Drug Design

**DOI:** 10.1155/ijm/2663212

**Published:** 2026-03-05

**Authors:** Leilei ZHAO, Jing QU, Miao AN, Yang LIU, Xiubo LI, Yiming LIU, Chen PENG

**Affiliations:** ^1^ Institute of Feed Research, Chinese Academy of Agricultural Sciences, Beijing, China, caas.cn; ^2^ Aplus Technology Innovation (Liaoning) Co. Ltd., Shenyang, China; ^3^ College of Veterinary Medicine, China Agricultural University, Beijing, China, cau.edu.cn

**Keywords:** computer-aided drug design, inhibitors, lumpy skin disease virus, mechanism of action, poxvirus

## Abstract

This review summarizes the research progress on inhibitors of lumpy skin disease virus (LSDV), focusing on its biological characteristics and key proteins involved in the viral replication cycle. It compiles various inhibitors (direct‐acting poxvirus inhibitors and host‐directed poxvirus inhibitors) against poxviruses, their mechanisms of action, and potential targets, aiming to provide references for the development of LSDV inhibitors. Additionally, the application of computer‐aided drug design methods in anti‐LSDV inhibitor research is discussed, offering insights from the perspective of virtual screening to facilitate the discovery of novel antiviral agents.

## 1. Introduction

Lumpy skin disease (LSD) is an acute or subacute infectious disease caused by the Lumpy skin disease virus (LSDV), which belongs to the *Poxviridae* family [1]. In recent years, LSD has been identified as one of the most devastating and emerging threats to large domesticated ruminants such as cattle, water buffalo, and wild bovine species [2]. It will seriously affect the healthy development of the global cattle industry and the international trade of livestock products [3]. LSD is classified by the World Organization for Animal Health (WOAH) as a notifiable transboundary disease. Interrupting viral transmission is crucial for mitigating disease impact. Given that vaccines cannot provide immediate protection, antiviral inhibitors present a promising emergency intervention. Identifying antiviral inhibitors that target LSDV is significant for enhancing the antiviral capacity of individual animals, alleviating symptoms of LSD, shortening disease duration, and reducing economic losses.

## 2. Biological Characteristics and Viral Replication Cycle of LSDV

LSDV, the smallpox virus, and the monkeypox virus are all classified under the *Chordopoxvirinae* (ChPV) subfamily of the *Poxviridae* family. Among them, LSDV, goatpox virus (GTPV), and sheeppox virus (SPPV) belong to the *Capripoxvirus* (CaPV) genus [4]. The pathogen is a large double‐stranded DNA virus with a linear genomic structure of approximately 151 kb, encoding about 156 open reading frames (ORFs). It exhibits typical poxviral morphological characteristics and biological properties [5].

Members of the *Poxviridae* family generally follow a highly conserved replication cycle, which typically includes key stages such as viral adsorption, cellular entry, uncoating, synthesis of viral genes and proteins, assembly and maturation of viral particles, and the final envelopment and release of the virus [6]. Classical antiviral drugs typically inhibit viral replication by blocking or interfering with key steps in the viral replication cycle. The entire replication process of LSDV occurs within the cytoplasm of the host cell. Its genome contains the majority of the functions necessary for independent replication, which characterizes it as a “self‐sufficient” virus. The life cycle of LSDV (Vero cell‐adapted) is 36–48 h in both Vero and MDBK cells [7]. The process of viral infection commences with the attachment of the virus to the cell surface. In the absence of specific viral receptors, viral particles bind directly to the cell membrane and enter the cell through various mechanisms. The virus enters the cell in two forms: the intracellular mature virus (IMV) and the extracellular enveloped virus (EEV). These forms are encapsulated by multiple membrane layers and contain specific proteins [8]. Gene expression occurs in three stages: early, intermediate, and late. Early genes are transcribed immediately after the virus enters the host cell, and their products primarily participate in viral DNA replication, transcriptional regulation, and evasion of the host immune system [9]. Many early proteins inhibit the host′s antiviral responses and remodel the intracellular environment to create favorable conditions for viral replication. The LSDV005, LSDV008, and LSDV142 genes expressed in the early stage of LSDV encode RNA polymerase, Kelch‐like protein, and anchor protein repeat domain protein, respectively. They play crucial roles in transcription, immune escape, and host interactions [10]. They are essential for establishing infection, functioning even before the initiation of viral DNA replication. Viral DNA replication begins after the completion of early gene expression [11]. LSDV, like other poxviruses, employs a rolling‐circle replication mechanism. The viral core undergoes a complex uncoating process, during which the core wall dissolves, releasing the viral genome into the cytoplasm. The genome then becomes enveloped by endoplasmic reticulum‐derived components and several early viral proteins, forming pre‐replication foci. As viral DNA replication intensifies, these foci expand into “virus foci”. Intermediate genes are expressed after the onset of DNA replication, and their products primarily contribute to late gene expression and the generation of factors required for virion assembly [12]. Late genes are expressed prior to virion assembly and encode structural proteins as well as enzymes essential for the formation of infectious virus particles [13]. The entry of LSDV into host cells relies on interactions between viral surface proteins and cellular receptors, though the specific host receptors involved in this process have not been clearly identified. Once the virus binds to a cellular receptor, it induces endocytosis, envelops the entire viral particle within an intracellular vesicle (endosome). The progressively acidifying environment within the endosome triggers viral uncoating, releasing the viral core structure into the cytoplasm.

### 2.1. Anti‐Poxviral Inhibitors and Targets

In recent years, with the improved understanding of poxviral biological structures and replication cycles, a number of highly effective and low‐toxicity anti‐poxvirus inhibitors have been gradually identified. Anti‐poxviral inhibitors typically target specific functions within the viral replication cycle. Functional proteins involved in viral replication and their related gene products are considered potential targets for drug development. Research has revealed a promising array of potential targets and inhibitors, which represent a promising foundation for developing new anti‐poxvirus drugs. Conventional antiviral discovery pipelines have primarily focused on agents that directly target viral proteins. However, strategies that disrupt host‐cellular factors essential for the viral life cycle are increasingly recognized for their potential to confer broad‐spectrum antiviral activity.

The Table 1 above summarizes approved or investigational inhibitors capable of suppressing poxviruses, functioned by direct‐acting poxvirus critical stages of the viral life cycle. Although such direct‐acting antiviral strategies offer high potency and specificity, their efficacy is often limited by the rapid emergence of drug‐resistant viral mutants. Consequently, strategies targeting host cellular factors, which are essential for viral replication yet genetically stable, present a compelling alternative. This host‐directed approach can provide a broader antiviral spectrum and a higher genetic barrier to resistance, thereby complementing direct antiviral strategies. Crucially, all members of a particular virus family often share a dependency on the same host cell signaling pathways to complete their replication cycle. For instance, members of the *Poxviridae* family, such as BPXV, LSDV, and VACV have been shown to critically rely on the host ROCK1 kinase signaling pathway for their replication [57]. All members of a particular virus family usually share the same kinase requirements. The key characteristics of these host‐targeting inhibitors are detailed in Table 2 below.

**Table 1:** Direct‐acting poxvirus inhibitors and their characteristics.

**Agent name**	**Category**	**Targeted virus**	**Experimental model**	**Mechanism/target**
Cidofovir (HPMPC) [14, 15]	Synthetic compounds‐nucleoside/nucleotide analogues	MPXV (monkeypox virus), VARV (Smallpox virus), CPXV (Cowpox virus), and other orthopoxviruses	In vitro: Vero and HFF (human foreskin fibroblast) cells In vivo: mice, rabbits, and monkeys	Inhibition of viral DNA synthesis at the level of viral DNA polymerase
Cyclic cidofovir (cHPMPC) [16]	Synthetic compounds‐nucleoside/nucleotide analogues	MPXV, VARV, CPXV, and other orthopoxviruses	In vitro: Vero cells (MA‐104, BSC‐40), HFF cells In vivo: mice, rabbits, and monkeys	Same as Cidofovir
Brincidofovir (CMX001) [17]	Synthetic compounds‐nucleoside/nucleotide analogues	MPXV, VARV, CPXV, and other orthopoxviruses	In vitro: Vero 76, LLC‐MK2 (Rhesus monkey kidney epithelial cells) and HFF cells In vivo: mice	Same as cidofovir
(S)‐HPMPA or octadecyloxyethyl‐(S)‐HPMPA [18, 19]	Synthetic compounds‐nucleoside/nucleotide analogues	MPXV, VARV, CPXV, and other orthopoxviruses	In vitro: Vero 76, LLC‐MK2 and HFF cells In vivo: mice	Same as cidofovir
N‐methanocarbathymidine [20]	Synthetic compounds‐nucleoside/nucleotide Analogues	VACV	In vitro: MA‐104 and LLC‐MK2 cells. In vivo: BALB/c mice	(N)‐MCT first undergoes phosphorylation in infected cells and then inhibits the viral DNA polymerase at the nucleoside triphosphate level
5‐substituted 4 ^′^‐thiopyrimidine nucleosides [21]	Synthetic compounds‐nucleoside/nucleotide analogues	VAC and CPXV	In vitro: HFF and Vero cells In vivo: BALB/c mice	Phosphorylation by the virus‐encoded thymidine kinase (TK) and inhibition of viral DNA synthesis
Arac [22, 23]	Synthetic compounds‐nucleoside/nucleotide analogues	VACV	In vitro: Hela cells In vivo: mice	Inhibition of viral DNA synthesis at the level of viral DNA polymerase
IUdR (IDU), EtUdR, NCSUdR [23]	Synthetic compounds‐nucleoside/nucleotide analogues	VACV	In vitro: HeLa cells In vivo: mice	Not detailed in this article
KAY‐2‐41 [24]	Synthetic compounds‐nucleoside/nucleotide analogues	VACV, CPXV, and CMLV	In vitro: HEL (human embryonic lung fibroblasts) cells In vivo: NMRI mice	Interaction ultimately with the viral DNA polymerase (E9L) to block virus replication. Partly relies on phosphorylation by the viral thymidine kinase (TK, J2R gene).
Ribavirin and mycophenolic acid [25]	Synthetic compounds‐nucleoside/nucleotide analogues	CMLV (camelpox virus), CPXV, MPXV, and VACV	In vitro: Vero 76, 3T3 and MA‐104 cells In vivo: mice	Inhibition of IMPDH and depletion of GTP
Ribavirin and tiazofurin [15, 26]	Synthetic compounds‐nucleoside/nucleotide analogues	CPXV, VACV, and other orthopoxviruses	In vitro: Vero 76, LLC‐MK2 cells In vivo: mice	Inhibits GMP biosynthesis IMP dehydrogenase, affects guanosine synthesis.
Trifluridine, and adefovir dipivoxil [27]	Synthetic compounds‐nucleoside/nucleotide analogues	VACV, MPXV, and CPXV	In vitro: HFF, HeLa, BSC‐1 and E6 cells	Inhibits viral DNA replication.
5,6‐dichloro‐1‐beta‐D‐ribofuranosylbenzimidazole (DRB) [28]	Synthetic compounds‐nucleoside/nucleotide analogues	VACV	In vitro: Ehrlich ascites tumor cells	Inhibits mRNA production in (uninfected) cells, presumably by causing a premature termination of RNA chains synthesized by RNA polymerase II.
Nitazoxanide (NTZ) [29]	Synthetic thiazolide antimicrobial drug	VACV	In vitro: HFF, BSC40, HeLa, and guinea pig fibroblasts cells	Impedes adaptations in cellular metabolism, particularly fatty acid metabolism.
ST‐246 (Tecovirimat) [30]	Synthetic compounds‐small molecule antiviral compounds	VACV, CPXV, and CMLV	In vitro: HEL, Vero, BSC‐1, and BHK‐21 cells	Inhibits the viral F13L phospholipase, thereby blocking the envelopment and egress of orthopoxviruses.
NIOCH‐14 [31]	Synthetic compounds‐small molecule antiviral compounds	ECTV (Ectromelia virus), MPXV, and VACV	In vivo: outbred ICR mice, Marmots, immunodeficient SCID mice	Same as ST‐246
Adenosine N1‐oxide (ANO) [32]	Synthetic compounds‐small molecule antiviral compounds	VACV	In vitro: conducted in cell culture, though the specific cell line is not stated. In vivo: BALB/c mice	Not explicitly detailed
Isatin‐*β*‐thiosemicarbazone and marboran [33]	Synthetic compounds‐small molecule antiviral compounds	VACV, CPXV	In vitro: HFF cells In vivo: BALB/c mice and SKH‐1 mice	Acts directly at the viral RNA polymerase complex during transcriptional elongation or indirectly by regulating elongation factors at the level of viral transcriptional termination.
Bisbenzamide Hoechst 33342 (H42) [34]	Synthetic compounds‐small molecule antiviral compounds	VACV, MPXV, TPOXX‐resistant VACV mutant (G277C)	In vitro: HeLa, A‐RPE‐19, BSC‐40, Hft, and Vero E6 cells	Inhibits viral DNA replication and subsequent late gene expression (LGE).
Rifampicin [35]	Synthetic compounds‐semisynthetic antibiotic	VACV	In vitro: BSC‐1 and CV‐1 cells	Transcription of the D13L gene was reduced and synthesis of the 65,000‐Da protein product was inhibited by more than 95%.
N,N ^′^‐bis(methylisatin‐beta‐thiosemicarbazone)‐2‐methylpiperazine [36]	Synthetic compounds‐small molecule antiviral compounds	VACV	In vitro: RK‐13 cells	Affects virus reproduction from 12 to 24 h postinfection.
PAV‐866 and other methylene blue derivatives [37]	Synthetic chemical substance	VACV, MPXV, and CXPV	In vitro: HeLa and BSC‐40 cells	Direct and irreversible inhibition of VACV virions prior to infection
Atovaquone, mefloquine, and molnupiravir [38]	Synthetic chemical substance	MPXV	In vitro: Vero E6, A549, and Huh‐7 cells	Atovaquone inhibits dihydroorotate dehydrogenase, mefloquine inhibits viral entry. Molnupiravir is a nucleoside analogue that targets polymerization of the genome of different virus classes.
CMLDBU6128 [39]	Synthetic compounds	VACV, MPXV, and CPXV	In vitro: A549, HeLa, Vero cells	Blocks postreplicative intermediate and late gene expression by targeting the viral RNA polymerase.
IMP‐1088 [40]	Synthetic compounds	VACV and MPXV	In vitro: A549 cell	Inhibits N‐myristoyltransferase (NMT), prevents the myristoylation of viral proteins (such as VACV L1R), thereby inhibiting viral cell entry
Nigericin [41]	Natural compound carboxylic ionophore	VACV	In vitro: HeLa, A549, Huh7, BHK21, and RK13 cells	As an ion carrier disrupts the ion gradient of host cells, the drastic changes in the cellular environment affect viral transcription and translation.
Distamycin A [42]	Natural product–derived small‐molecule compounds	VACV	In vitro: BSC40 and Hela cells	Represses transcription of intermediate genes encoding late transcription factors (e.g., A1L, A2L, and G8R), and directly inhibits late promoter activity, preventing late protein synthesis and virion assembly.
4‐bromo‐N ^′^‐((1R,4R)‐1,7,7‐trimethylbicyclo[2.2.1]heptan‐2 ylidene)benzohydrazide 18 [43]	Natural product derivatives	VARV, CPXV, and ECTV	In vitro: Vero cells	Remains unknown
Apigenin [44]	Natural product derivatives	BPXV	In vitro: Vero cells In ovo: embryonated chicken eggs	Inhibits synthesis of viral DNA, mRNA, and proteins, inhibits the ERK/MNK1/eIF4E signaling pathway.
Sansalvamide A [45]	Marine natural peptides	MCV (Molluscum contagiosum virus)	In vitro: cell‐free biochemical assays or enzyme activity measurements	Inhibition of DNA binding by the MCV topoisomerase, thereby preventing the formation of the enzyme‐DNA covalent complex
Contact‐blocking Viral Inhibitor (CVI) [46]	Natural heat stability	VACV	In vitro: mouse L929 cells	Prevents virus attachment to host cells, thereby blocking the initial step of infection.
Salvia officinalis extract [47]	Natural product derivatives	VACV	In vitro: HeLa cells	Inhibits viral topoisomerase I encoded by the poxvirus, thereby blocking viral replication.
Glycyrrhizic acid [48]	Natural product derivatives	VACV	In vitro: HEP‐2 cell line	May interacts with viral proteins (still a hypothesis).
Ethanol extracts from *Bignoniaceae* plants [49]	Natural product derivatives	VACV	In vitro: Vero cells	Not specified
Marine sulfated glycans (Sulfated fucans, Fucosylated chondroitin sulfates) [50]	Natural product derivatives	MPXV	In vitro: SPR	Competitively inhibits viral protein binding to host cell surface heparan sulfate. Targets the virus‐host interaction.
Sodium magnesium‐chlorophyllin [51]	Natural product derivatives	VACV	In vitro: direct observation of virus particles by electron microscopy, with no cell lines or animal models involved.	Acts by directly destroying the virion membrane and subsequently leading to destruction of the viral nucleic acid, thereby inactivating the virus.
The aqueous extract of *Quillaja saponaria* Molina [52]	Natural product derivatives	VACV	In vitro: human 143 cells	Blocks virus attachment to host cells by modifying the cell membrane or virus receptors, preventing infection.
Zinc oxide nanoparticles (ZnO‐NPs) [53]	Inorganic metal nanomaterials	MCV	In vitro: BHK‐21	Reactive oxygen species (ROS) production and hydrogen peroxide (H₂O₂) formation: which can inactivate MCV through viral protein oxidation or viral genome destruction. blocks viral entry and spread.
Multivalent 2D nanosystems [54]	Synthetic nanomaterials	VACV, CPXV, and MPXV	In vitro: Vero E6 and Hep2 cells	Competitively binds to viral A27 protein via sulfate groups, mimicking heparan sulfate and blocking viral attachment to host cells.
SiO₂‐AZT∗TP [55]	Nucleoside analogue prodrug (AZT triphosphate analogue) delivered by SiO₂ nanoparticles	Poxvirus (specifically, the POX virus, strain K‐1, which is a mouse smallpox virus)	In vitro: Vero cells	Acts as a DNA chain terminator. The delivered AZT triphosphate analogue is incorporated by viral DNA polymerase into the growing DNA chain but prevents further elongation due to its modified 3 ^′^‐end (triazole group), thereby terminating viral DNA replication.
Nanohybridized niclosamide [56]	Nanohybridized drug formulation	MPXV	In vivo: Syrian hamster model	Blocks viral entry by targeting viral envelope proteins, replication associated with the inhibition of mTOR and Wnt/*β*‐catenin, reduces the excessive inflammatory response through NF‐*κ*B Pathway, STAT3 Pathway and NLRP3 Inflammasome.

**Table 2:** Host‐directed poxvirus inhibitors and their characteristics.

**Agent name**	**Category**	**Targeted virus**	**Experimental model**	**Mechanism/target**
Carbocyclic 3‐deazaadenosine (C‐c3‐Ado), 3‐deazaneplanocin A (C3‐Npc A) [15, 58, 59]	Synthetic compounds‐small molecule antiviral compounds	MPXV, VARV, VACV, and other orthopoxviruses	In vitro: Vero 76, LLC‐MK2, PRK (Primary rabbit kidney) cells In vivo: mice	Inhibits SAH hydrolase and blocks viral methylation.
Ibrutinib [60]	Synthetic compounds‐small molecule antiviral compounds	VACV, MPXV, and LSDV	In vitro: HeLa, A549, THP‐1, HFF, Vero, and DF‐1, MDBK cells In vivo: BALB/c mice	Inhibits the phosphorylation and nuclear translocation of the host cellular BTK, thereby suppressing poxviral DNA replication and postreplicative gene expression, while also priming the Type I interferon response.
Nitroxoline [61]	Synthetic compounds‐small molecule antiviral compounds	MPXV	In vitro: HFF, HFK, ARPE, and HaCaT cells	Inhibits host cell signaling pathways (PI3K/AKT/mTOR and RAF/MEK/ERK).
Imatinib(STI‐571/Gleevec) [62]	Synthetic compounds‐small molecule antiviral compounds	VACV	In vitro: 3T3, BSC‐40 cells In vivo: C57BL/6 mice	Targets the Abl‐family tyrosine kinases in host cells, inhibits release of infectious EEV.
Canertinib(CI‐1033) [63]	Synthetic compounds‐small molecule antiviral compounds	VACV	In vitro: Vero, BSC‐1, and Hela cells	Binds to the Cys773 residue of ErbB‐1 (EGFR) covalently, permanently inhibits its kinase activity.
Aurintricarboxylic acid (ATA) [64]	Synthetic compounds‐small molecule antiviral compounds	VACV	In vitro: HeLa, Huh7, AD293, RK13, Vero, 3T3, and BHK21 cells	ATA blocks the phosphorylation of extracellular signal‐regulated kinase, and inhibits the phosphatase activity of the viral enzyme H1L.
Smallpox growth factor mimics [64]	Synthetic compounds‐small molecule mimics	VARV	In vitro: BSC‐40 and Vero‐E6 cells In vivo: C57BL/6 mice	Binds to and inactivates Erb‐B1, a receptor tyrosine kinase of importance to vaccinia virus replication.
IMP‐1088 [40]	Synthetic N‐myristoyltransferase (NMT) inhibitor	VACV and MPXV	In vitro: A549 cell	Inhibits N‐myristoyltransferase (NMT), prevents the myristoylation of viral proteins (such as VACV L1R), thereby inhibiting viral cell entry.
SB239063 [65]	Systematic compounds	BPXV	In vitro: Vero	Targets the p38–MNK1–eIF4E signaling pathway.
CGP57380 [66]	Synthesized small‐molecule inhibitors	BPXV	In vitro: Vero and HeLa cells In ovo: chicken embryo	Targets the ERK/MNK1/eIF4E signaling pathway, disruption of eIF4E/eIF4G interaction
4EGI‐1^66^	Synthesized small‐molecule inhibitors	BPXV	In vitro: Vero and HeLa cells In ovo: chicken embryo	Disruption of eIF4E/eIF4G interaction, MNK1 regulates BPXV protein synthesis
Thiazovivin, Y27632, fasudils, and GSK269962A [67, 68]	Synthesized small‐molecule inhibitors	BPXV, VACV	In vitro: Vero and HeLa cells In ovo: embryonated chicken eggs	Targets the ROCK1/MLC‐2 signaling pathway, inducing the decay of poxviral viral mRNA in virus infected cells
Ibrutinib	Systematic compounds‐selective Bruton tyrosine kinase (BTK) inhibitor.	VACV, MPXV, and LSDV	HeLa, A549, THP‐1, HFF, Vero, DF‐1, MDBK cells In vivo: BALB/c mice	Inhibits the phosphorylation and nuclear translocation of the host cellular BTK, thereby suppressing poxviral DNA replication and postreplicative gene expression, while also priming the Type I interferon response.
Silver nanoparticles [69]	Synthetic nanomaterial‐based agents	VACV	In vitro: VERO 76, BS‐C‐1, and HeLa cells	Inhibits viral entry by blocking micropinocytosis.
Terameprocol (TMP) [70]	Semi‐synthetic compound, Phenolic antioxidant	CPXV and VACV	In vitro: 143B, BS‐C‐1, 293, Hep‐G2, A431, CCD‐1138SK, and C3HA	TMP interferes with the formation of actin tails, Inhibiting the host transcription factor C/EBP*β* can specifically shut down viral late gene expression.
Interferon and polyacrylic acid [71]	Interferon: Natural immunomodulatory protein. Polyacrylic acid: chemically synthesized. Synthetic polymer	VACV	In vivo: NMRI mice	Interferon: Induces an antiviral state in cells, thereby inhibiting viral replication. Polyacrylic acid (PAA): Induces the production of interferon, which indirectly mediates its antiviral effect. The paper discusses that its protective effect is closely related to its interferon‐inducing ability.
Cardiac glycosides [72]	Natural steroid glycosides	VACV	In vitro: HeLa, BSC‐40, and A‐RPE‐19 cells	Inhibits early and late vaccinia virus protein expression as the cellular sodium‐potassium ATPase pump inhibitor.
Mitoxantrone [73]	Natural anticancer drug	VACV	In vitro: BSC‐40 cells	Blocks processing of viral structural proteins and assembly of mature progeny virions.
Novobiocin [74]	Natural antibiotic	VACV	In vitro: BSC‐40 cells	Inhibits a very early stage of morphogenesis, so that membrane crescents and spherical immature virions do not accumulate.
Cyclosporin A (CsA) [75]	Natural cyclic undecapeptide of fungal origin.	VACV	In vitro: BSC‐40 and Balb/c fibroblasts cells	Inhibits viral DNA synthesis, which in turn affects late viral protein synthesis.
Prostaglandins of the A series [76]	Naturally occurring cyclic 20‐carbon fatty acids	VACV	In vitro: mouse L fibroblasts cells	Delays and partially inhibits virus DNA synthesis through host signaling pathways.
Nigericin [41]	Natural compound carboxylic ionophore	VACV	In vitro: HeLa, A549, Huh7, BHK21, and RK13 cells	As an ion carrier disrupts the ion gradient of host cells, the drastic changes in the cellular environment affect viral transcription and translation.
Hesperetin [77]	Naturally occurring flavonoid	BPXV, VACV, and LSDV	In vitro: Vero In ovo: SPF chicken embryo	Competitively binds to the mRNA 5 ^′^cap‐binding pocket of eIF4E, preventing viral mRNA from initiating translation
Emetine [78]	Natural alkaloid	BPXV	In vitro: Vero and MDBK cells In ovo: embryonated chicken eggs	The target is the ribosome, inhibiting protein synthesis in host cells.

Emetine treatment did not lead to observable resistance development after 25 passages [78]. No drug‐resistant virus variants were detected following 50 serial passages of BPXV under the pressure of the ROCK1 inhibitor Thiazovivin [67]. Although host‐directed antiviral agents are generally associated with a higher genetic barrier to resistance, our findings demonstrate that this property is not absolute. Specifically, we observed that buffalopox virus (BPXV) could develop partial resistance (~tenfold) to the MNK1 inhibitor CGP57380 after 40 passages under drug pressure. This indicates that while the emergence of resistance is considerably slower and less pronounced compared with direct‐acting antivirals, it does not completely preclude the eventual selection of less susceptible viral variants under sustained, high‐concentration challenge [66]. After 60 passages, Chander et al. [65] successfully isolated the BPXV‐P60‐SB239063 virus, which exhibited markedly enhanced replication under SB239063 treatment compared with the control virus (~sixfold–~hundredfold inhibition), demonstrating the development of partial resistance. Contrary to the notion of absolute resistance evasion, this study demonstrates that viral resistance to host‐directed antivirals can emerge through the adaptation of utilizing alternative host factor isoforms (e.g., a switch from p38‐*α* to p38‐*γ*) under sustained selective pressure, thereby establishing that the genetic barrier to resistance, whereas high, is not insurmountable [11]. The above research provides significant reference value for the development of anti‐LSDV inhibitors. Drug repurposing serves as an effective approach to shorten the drug development cycle. Given the high genetic sequence similarity of key proteins among viruses in the *Poxviridae* family, it is plausible that inhibitors effective against poxviruses will extend their efficacy to LSDV.

## 3. Anti‐LSDV Inhibitors and Targets

In cows naturally infected with LSD, administration of acyclovir and ivermectin (IVM) accelerated the recovery rate and significantly reduced clinical symptoms [79]. Hesperidin, a natural flavonoid found in citrus fruits, grapes, lemons, and other fruits, has demonstrated antiviral activity against poxviruses including LSDV, buffalo pox virus (BPXV), and vaccinia virus (VACV). This research has shown that hesperetin blocks viral protein synthesis by inhibiting the binding of eukaryotic initiation factor eIF4E to the 5 ^′^ cap structure of viral mRNA [77]. Toker et al. [79] investigated the antiviral efficiency of IVM against LSDV and SPPV at different stages of in vitro replication. The mean viral titers during the replication stages of LSDV and SPPV were significantly reduced by approximately three logarithms (*p* < 0.05). IVM influenced the CaPV replication cycle during postentry stages more effectively, such as viral replication. The small‐molecule inhibitor of Class IIa histone deacetylases, TMP269, has been demonstrated to suppress the early‐stage replication of LSDV in a dose‐dependent manner [80]. Its antiviral effect involves interfering with LSDV‐induced alterations in host glycerophospholipid metabolism, notably by reducing the infection‐driven elevation of the key metabolite lysophosphatidic acid (LPA). Further studies suggest that LSDV infection may enhance LPA expression, which activates the MEK/ERK signaling pathway and concurrently dampens the host innate immune response, creating a cellular environment conducive to viral proliferation. In contrast, TMP269 counteracts this mechanism. These insights offer a new framework for understanding LSDV pathogenesis and for designing antiviral approaches focused on modulating host metabolism. Wang et al. [81] constructed a recombinant virus LSDV‐*Δ*TK/EGFP expressing enhanced green fluorescent protein (EGFP) for high‐throughput screening of antiviral drugs. They found that gallic acid, emodin, theaflavin, 4‐ethylphenol, and narcissin strongly inhibited LSDV during both cellular entry and subsequent viral replication stages. Among these, gallic acid, emodin, and theaflavin demonstrated superior efficacy. Victor P[82] evaluated the antiviral activity of n‐hexane, dichloromethane, acetone, and methanol extracts from six traditional anti‐infection plants against canine distemper virus (CDV), canine parainfluenza virus‐2 (CPIV‐2), feline herpesvirus‐1 (FHV‐1), and LSDV in vitro. The acetone and methanol extracts from *Podocarpus macrophyllus* exhibited the strongest inhibitory effects against CDV and LSDV, suppressing viral replication by over 75% at a concentration of 3 *μ*g/mL.

LSDV contains most of the conserved poxvirus genes involved in fundamental replication mechanisms. Previous studies have confirmed that numerous ORFs are either upregulated or downregulated during specific stages. Additionally, time‐of‐addition assays serve as one of the key experimental approaches for investigating inhibitor mechanisms of action. The time‐of‐addition assay for LSDV inhibitors involves introducing inhibitors and viruses into host cells separately or simultaneously under varying temperature conditions and at specific time points. This method examines the degree of viral titer suppression at different stages of the viral replication cycle and monitors changes in gene expression. It verifies the impact on key proteins at distinct stages of gene expression, serving as a crucial approach for elucidating LSDV′s mechanism of action and identifying potential targets. The Neethling strain of LSDV initiates early mRNA synthesis immediately upon infection and lasts for approximately 9 h. DNA replication commences around 10 h postinfection, transitioning into late transcription. If DNA synthesis is inhibited, late genes remain silenced and are only activated upon replication completion [82]. When exploring the mechanism of anti‐LSDV inhibitors, researchers often rely on time‐of‐addition experiments as an important indirect method for target identification. After determining the stage of action, suspected targets are subsequently validated using methods such as qPCR, Western blot, or ELISA.

The current status of research on LSDV inhibitors and target validation data is summarized in Table 3 below.

**Table 3:** Summary of LSDV inhibitors by action stage and potential targets.

**The two main strategies for developing antivirals**	**Inhibitor**	**Category**	**Stage of action–time-of-addition assay**	**Validated potential targets**
Host‐directed antiviral agents	Hesperidin [77]	Naturally occurring flavonoid	Late gene transcription stage	Inhibits viral late gene expression; multiple common targets were ruled out, narrowing the focus to a highly conserved viral process related to the viral transcription mechanism.
Host‐directed antiviral agents	Ivermectin [79]	Semisynthetic drug	Strongest inhibitory effect during the replication stage; moderate inhibitory effect during the early stages (adsorption and entry)	Proposed as a nuclear import inhibitor (via inhibition of importin *α*/*β*1)
Host‐directed antiviral agents	TMP269 [80]	Chemically synthesized	Early stage of the viral life cycle	Focus on host targets and metabolic pathways; modulates host cell glycerophospholipid metabolism involving lysophosphatidic acid and lysophosphatidylcholine.
Host‐directed antiviral agents	Theaflavin [81]	Natural product	Viral entry into cells and subsequent replication stages	LSDV ORF142 protein and validated at the gene level
Direct‐acting antiviral therapy	Acetone and methanol extracts of *Podocarpus henkelii* [83]	Natural extract	Inactivates virus prior to adsorption	Acts on the viral envelope
Direct‐acting antiviral therapy	Ibrutinib [16]	Chemically synthesized	Partially inhibits early gene expression; significantly inhibits intermediate and late gene expression; reduces DNA replication by 50%	E3L mRNA and early proteins (E3, H5, and I3) partially reduced; D13L and A3L mRNA and late protein A3L significantly reduced; E11L (DNA replication) reduced

The data in the table can provide valuable reference for the development of LSDV, SPPV, and GTPV inhibitors. And also, the targets or mechanisms of LSDV show reference value for both SPPV and GTPV. Before the accessibility of full genome LSDV sequences, all CaPVs came into being by recombination from a single ancestor [84]. SPPV and GTPV share antigenic homology and cross protection with LSDV, they share more than 97% similarity with their viruses [85].Besides this, research into the LSDV replication cycle has identified other key proteins that represent promising potential targets for investigation. These include DNA polymerase (LSDV039) and DNA topoisomerase (LSDV077). Additionally, the LSDV‐encoded proteins LSDV145 and LSDV147 may be involved in suppressing virus‐induced apoptosis [86]. Xie et al. [87] discovered that LSDV012, a host‐specific immune evasion protein, promotes viral replication by targeting and inhibiting the antiviral function of the host protein IFIT1. This mechanism is one of the key strategies LSDV employs to achieve immune evasion and determine its host range. The results made by Zhao [88] are the first to show that SPPV infection induces phosphorylation of eIF2 through PKR activation, which then results in restriction of CaPV replication. GTPV K3L strongly inhibited sPKR and goat PKR (gPKR), but SPPV K3L only partially inhibited gPKR. The partially purified 35 kDa protein from the fecal matter of silkworms (*Bombyx mori*) demonstrates significant hepatoprotective effects and in vitro antioxidant activity, but it does not exhibit inhibitory effects against camelpox virus or GTPV under the tested conditions [89].

Although research on poxvirus inhibitors has encompassed a wide range of substances and formulations—including nucleoside analogues, synthetic compounds, natural products, marine extracts, nanomaterials, and carbon‐based materials—the development of specific inhibitors against LSDV remains in its early stages, with relatively limited options available. Both direct‐acting antiviral strategies and host‐directed approaches should be concurrently pursued and applied to enhance antiviral efficacy and reduce the risk of drug resistance. VACV, as a model poxvirus, provides a valuable reference for LSDV drug discovery, as inhibitors effective against vaccinia often exhibit activity against LSDV. Moving forward, anti‐LSDV inhibitor research should expand into more diverse material systems and actively incorporate novel excipients and advanced materials (e.g., carbon‐based structures, nanocarriers). The application of formulation technologies such as nano‐liposomes and dispersions holds promise for significantly enhancing drug‐delivery efficiency and therapeutic efficacy, thereby providing robust support for the development of anti‐LSDV pharmaceuticals.

## 4. Drug Discovery Methods and Computer‐Aided Drug Design

Drug discovery efforts can be conducted in laboratory environments or through computer‐aided drug design. The resurgence of orthopoxvirus outbreaks has drawn widespread attention once again. To address the challenges posed by these viruses, we cannot rely solely on traditional drug discovery processes to identify novel therapies. Virtual screening and bioinformatics‐based approaches, which can be used to repurpose or modify existing drugs, represent one of the most impactful and cost‐effective methods. To accelerate the discovery of LSDV‐resistant candidates, researchers have drawn upon experience from monkeypox drug screening and introduced computer‐aided methods such as virtual screening into the early stages of the R&D process.

Chen et al. [90] predicted the protein structure of the monkeypox F13 gene product (VP37 protein) and its potential binding pockets first. Then they performed virtual screening of 10,640 compounds from traditional Chinese medicine, experimental drug, and approved drug libraries against the protein encoded by F13. The study identified glecaprevir, dolutegravir, bictegravir, tucatinib, ubujil, zk‐806, protohypericin, hypericin, and ginkgetin as promising candidates for repurposing to treat MPXV. Akash et al. [91] identified curcumin derivatives as potential antiviral agents against monkeypox and smallpox viruses. They conducted molecular docking experiments with 50 different curcumin derivatives; 12 derivatives even demonstrated higher binding scores than the standard drug. Furthermore, all evaluated derivatives showed excellent ADMET (absorption, distribution, metabolism, excretion, and toxicity) properties. Krishna et al. [92] demonstrated through integrated ADMET prediction, molecular docking, and molecular dynamics simulations that apigenin‐4 ^′^‐glucoside and veracovir are promising antiviral inhibitors against LSDV. Zia et al. [93] highlighted the significant potential of drug repurposing and plant‐derived compounds as therapeutic candidates for LSD. Among 380 plant‐derived compounds evaluated, emodin and quercetin emerged as the most effective binders to the LSDV‐encoded DNA polymerase (LSDV039), characterized by their strong binding affinity. Among 718 FDA‐approved antiviral drugs, canagliflozin and tepotinib exhibited higher binding affinity.

Although the complete genome sequencing of LSDV has been accomplished, there remain several hypothetical genes with unknown functions, which may play key roles in disease pathogenesis and serve as targets for drug discovery. This study is aimed at exploring the LSDV004 gene using bioinformatic tools to predict its key properties, structure, functions, and potential inhibitors. This provides a theoretical foundation for its potential as a novel drug target or vaccine candidate [94]. Byrd et al. [95] conducted a computational screening on a chemical library containing approximately 51,000 compounds targeting the I7L protease of VACV. Through a series of methods including phenotypic analysis, electron microscopy observation, drug‐resistant virus screening, and gene localization, as well as gene rescue experiments, the study confirmed that I7L is the drug′s target. A class of small‐molecule compounds (represented by TTP‐6171) has been discovered.

## 5. Summary and Outlook

This review summarizes the research progress on various antiviral inhibitors targeting LSDV and poxviruses. Recent researches on anti‐poxvirus agents have focused on structural optimization of marketed and investigational drugs, exploration of novel targets, and design of corresponding inhibitors. Through prodrug modification, drug repurposing, compound library construction, and development of traditional Chinese medicine components, novel inhibitors are continuously discovered via phenotypic and virtual screening, with the binding sites and mechanisms of some inhibitors elucidated. Researches on poxvirus inhibitors provides crucial references for LSDV drug development. Studies on inhibitor antiviral activity at in vivo, in vitro cellular, and computational levels, along with phase studies through drug addition experiments and validation of expressed genes or targets, all hold significant reference value.

Currently, most inhibitor screening is based on “phenotypic screening” or “drug repurposing.” There is an urgent need to deepen the investigation into the targets and molecular mechanisms of LSDV inhibitors. In particular, advanced techniques such as X‐ray crystallography and cryo‐electron microscopy should be employed to analyze and characterize the interaction patterns between key viral proteins (such as DNA polymerase, viral membrane proteins, and viral factors) and inhibitors, as well as the three‐dimensional structures, binding sites, and conformational dynamics of drug‐protein complexes.

The goal is to develop novel small‐molecule inhibitors or protein–protein interaction inhibitors with high specificity and affinity. This will fundamentally enhance drug efficacy and specificity while reducing off‐target toxicity.

## Disclosure

All authors have read and agreed to the published version of the manuscript, and agreed to be accountable for the content and conclusions of this article.

## Conflicts of Interest

The authors declare no conflicts of interest.

## Author Contributions

Leilei ZHAO: conceptualization, methodology, writing—original draft, project administration. Jing QU: investigation, Data Curation, writing—review and editing. Miao AN: formal analysis, visualization, writing—review and editing. Yang LIU: data validation, writing—review and editing. Xiubo LI: validation, supervision. Yiming LIU: supervision, resources, writing—review and editing. Chen PENG: supervision, writing—review and editing, funding acquisition. Yiming LIU and Chen PENG are co‐corresponding authors.

## Funding

This study was supported by the National Key Research and Development Program of China (10.13039/501100012166, No. 2021YFD1800700).

## Data Availability

Data sharing is not applicable to this article as no datasets were generated or analyzed during the current study.
